# MPRAP: An accessibility predictor for *a*-helical transmem-brane proteins that performs well inside and outside the membrane

**DOI:** 10.1186/1471-2105-11-333

**Published:** 2010-06-18

**Authors:** Kristoffer Illergård, Simone Callegari, Arne Elofsson

**Affiliations:** 1 Center for Biomembrane Research, Stockholm Bioinformatics Center, Dept. of Biochemistry and biophysics, Stockholm University, SE-106 91 Stockholm, Sweden

## Abstract

**Background:**

In water-soluble proteins it is energetically favorable to bury hydrophobic residues and to expose polar and charged residues. In contrast to water soluble proteins, transmembrane proteins face three distinct environments; a hydrophobic lipid environment inside the membrane, a hydrophilic water environment outside the membrane and an interface region rich in phospholipid head-groups. Therefore, it is energetically favorable for transmembrane proteins to expose different types of residues in the different regions.

**Results:**

Investigations of a set of structurally determined transmembrane proteins showed that the composition of solvent exposed residues differs significantly inside and outside the membrane. In contrast, residues buried within the interior of a protein show a much smaller difference. However, in all regions exposed residues are less conserved than buried residues. Further, we found that current state-of-the-art predictors for surface area are optimized for one of the regions and perform badly in the other regions. To circumvent this limitation we developed a new predictor, MPRAP, that performs well in all regions. In addition, MPRAP performs better on complete membrane proteins than a combination of specialized predictors and acceptably on water-soluble proteins. A web-server of MPRAP is available at http://mprap.cbr.su.se/

**Conclusion:**

By including complete *a*-helical transmembrane proteins in the training MPRAP is able to predict surface accessibility accurately both inside and outside the membrane. This predictor can aid in the prediction of 3D-structure, and in the identification of erroneous protein structures.

## Background

During the folding of a protein some residues become exposed to the environment while others become buried in the protein interior. For water-soluble proteins the dominant driving force during folding is the hydrophobic effect, which minimizes unfavorable interactions between hydrophobic residues and (hydrophilic) water [1,2]. Therefore, water-soluble proteins consist of a hydrophobic interior and hydrophilic exterior. In contrast, the tendency to bury polar residues from the (hydrophobic) solvent environment within the membrane is much weaker. In membrane proteins residues face three distinct environments; a hydrophobic lipid environment inside the membrane, a hydrophilic water environment outside the membrane and an interface region in between. Studies of the bacteriorhodopsin structure suggested that membrane proteins are "inside-out", i.e. that they consist of a hydrophilic interior and a hydrophobic exterior [3-7]. However, later studies indicated that the "inside-out" rule is not generally applicable [6-10]. Since membrane proteins are exposed to distinctly different environments, the composition of exposed residues will differ significantly in different regions. Also, the main driving forces of folding and stabilization are different from water-soluble proteins and less well understood. However, irrespectively of environment, buried residues are in general under stronger evolutionary constraints than exposed sites [11].

For membrane proteins most bioinformatical efforts have been focused on the development of methods to predict the topology, i.e. the location of residues relative to the membrane. A topology prediction might be a useful first step towards structure prediction, while a predictor of solvent accessibility provides complementary information. Such a predictor might also be useful for predicting functional relevance of individual residues, since residues responsible for e.g. catalysis or substrate binding, are often buried in the protein interior [11], while residues involved in protein-protein-interactions occur on solvent exposed sites. For water-soluble proteins many methods for predicting the accessibility have been developed [12].

However, only a few attempts have been made to predict the accessibility of membrane proteins [9,13-15]. To our knowledge all existing methods have been specialized to predict the exposure within the membrane. Therefore, these methods require an initial prediction step to determine the exact location of the transmembrane segment, which current topology predictors only can do with a limited accuracy [16]. Here, we constructed a single accessibility predictor for entire membrane proteins. First, the amino acid distributions and evolutionary conservation in a set of *α*-helical transmembrane proteins of known structures were analyzed. Thereafter, we examined the ability of state-of-the-art predictors to identify exposed and buried residues. In particular, we analyzed the performance of the predictors in regions for which they had been optimized as well as of the regions where they had not. Subsequently, we developed a novel predictor, MPRAP, optimized to perform well in all regions. Finally, we investigated some additional potential uses of MPRAP.

## Results and Discussion

### Membrane protein surfaces adapt to the environment

**Figure 1:**
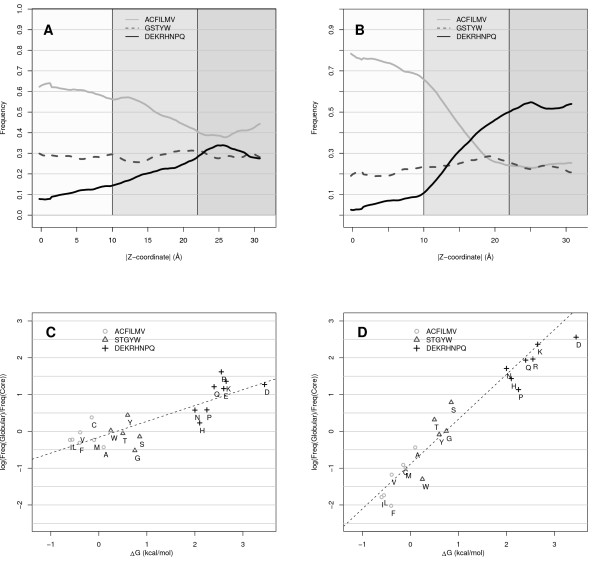
**Amino acid distribution and solvent accessibility**. The incidence of three different classes of amino acids as a function of distance from the membrane center. A) The subset of residues that have accessibility lower or equal to 25% (buried). B) The subset of residues that have accessibility higher than 25% (exposed). The coloring differentiates between polar, intermediate and hydrophobic residues. The two lower figures show the log of the difference in substitution rates between the globular and membrane regions plotted against the biological hydrophobicity value for each amino acid type. In C) the rate for buried sites is shown while in D) the rate for exposed sites is shown.

**Figure 2:**
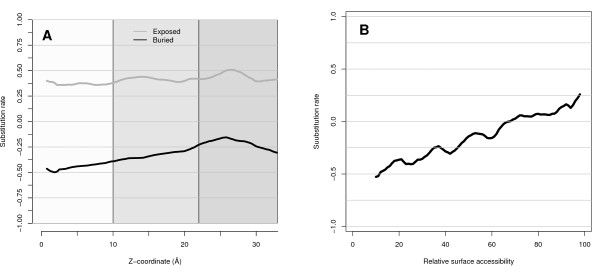
**Relative substitution rate and solvent accessibility**. A) The relative substitution rate as a function of Z-coordinate. Evolutionarily conserved sites have low values and variable sites have high independently on their distance from the membrane center. Sites that are solvent accessible and inaccessible are colored differently. B) The relative substitution rate as a function of relative accessibility for all residues show a linear relationship.

All residues in the dataset were classified by their distance from the membrane center into non-membrane (>22 Å from the membrane center), lipid-water interface (10-22 Å) or membrane core (<10 Å). Alternatively, residues were classified to be within or outside the membrane using the membrane boundaries from OPM [17]. Further, residues were divided into two accessibility classes, by using a cutoff of 25% exposed accessible surface area. Outside the membrane solvent exposed sites consist of 19% hydrophobic residues (A, F, I, L, M and V), while buried sites consist to 49% of such residues, see Figure 1. Thus, as in soluble proteins, the non-membrane regions of *α*-helical transmembrane proteins have a hydrophilic exterior and a hydrophobic interior. The membrane exposed sites consist of 73% hydrophobic residues and buried of 60%. This means that both exposed and buried sites are more hydrophobic inside the membrane, but the difference for buried sites is much smaller, see Figure 1C and 1D. Another observation is that the solvent accessibility and selective pressure is tightly connected; Buried residues are replaced at a slower rate than exposed residues in all solvent environments, Figure 2A[11]. Actually, the relative substitution rate is approximately linearly related to the accessibility, Figure 2B. This supports earlier observation that substitution rates might be useful for identifying exposed residues in membrane proteins [18].

### Surface area predictors optimized for one of the environments performs badly in the other environments

Quite a few methods for predicting accessibility of soluble proteins have been developed in the past [12]. Due to the low number of determined membrane protein structures most methods have been developed primarily for water-soluble proteins. However, there exist a few methods that have been developed for membrane proteins, including BW [13], LIPS [9], *ASAP*_*mem *_[14] and TMX [15]. Here, we investigated the performance of the two most recent membrane predictors, *ASAP*_*mem *_and TMX, as well as three recent predictors for soluble proteins, *ASAP*_*glob *_[14], SABLE [19] and ACCPRO [12]. The ability to accurately predict the accessibility state of residues in membrane proteins was examined. The predictors differ in their output; ACCPRO and TMX predict accessibility in a binary alphabet, i.e. exposed and buried, with an approximately equal fraction in both classes, while SABLE, *ASAP*_*glob*_, and *ASAP*_*mem *_predict the relative accessibility. Therefore, for comparisons the real value predictions were transformed to binary states. The specific cutoffs for transformations were optimized for each method independently, resulting in an approximately equal frequency of buried and exposed residues.

**Figure 3:**
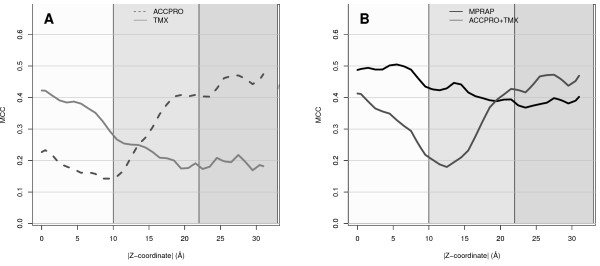
**Predicting buried residues**. Performance for predicting buried residues at different distances from the membrane center. A) A predictor for membrane region, TMX, and a predictor for soluble proteins, ACCPRO. B) The novel predictor MPRAP is compared to the combination of TMX and ACCPRO.

We found that the best method in the membrane region is the membrane specific predictor TMX (MCC = 0.32), and the best method in the non-membrane region is the water-soluble predictor ACCPRO (MCC = 0.47), see Table 1. However, TMX performs badly in the non-membrane region (MCC = 0.14) and ACCPRO performs badly inside the membrane (MCC = 0.20). Thus, the surface area predictors optimized for one of the environments performs badly in the other environment, see Figure 3. From these results a reasonable solution would be to use a combination of a membrane predictor and a soluble predictor. To test such an approach we used Zpred [20], a predictor of the distance from membrane center, together with TMX and ACCPRO. After optimization it was found that the best performance was obtained using TMX if a residue was predicted to be closer than 12.5Å from the membrane center and ACCPRO for all other residues. We found that the combination performed acceptable in the membrane core (MCC = 0.33) and well in the soluble region (MCC = 0.53). However, within the water-lipid interface region the combination performs suboptimally, most likely due to that none of the predictors were optimized for this region (MCC = 0.30), see Figure 3.

**Table 1:** Benchmarking accessibility predictors

	Mem Proteins						W-S Proteins
	All	Z:0-10	Z:10-22	Z: > 22	Membr	non-Membr.	All
MPRAP	0.45	0.47	0.40	0.49	0.45	0.46	0.55
ACCPRO	0.41	0.19	0.34	0.53	0.20	0.47	0.63
SABLE	0.29	0.08	0.24	0.46	0.11	0.38	0.55
TMX	0.21	0.35	0.19	0.13	0.32	0.14	-
ACCPRO+TMX	0.39	0.33	0.30	0.53	0.31	0.47	-
*ASAP*_*mem*_	0.12	0.05	0.14	0.11	0.08	0.13	-
*ASAP*_*extmem*_	0.40	0.26	0.41	0.47	0.27	0.44	-
*ASAP*_*glob*_	0.32	0.11	0.25	0.46	0.11	0.36	-

A comparison of the performance for different accessibility predictors using two-state predictions in water-soluble (W-S) proteins and membrane (Mem) proteins. The reported values are the Matthew correlation coefficients for identifying buried residues in a binary alphabet. Analysis was performed the entire protein (whole) or regions in the membrane either divided by Z-coordinate or by membrane definitions from OPM. Due to computational limitations only three predictors were applied on the water-soluble dataset.

### Optimization of MPRAP

To overcome the problems with the environment specialized prediction methods we developed a novel Support Vector Machine (SVM) based predictor. It was named Membrane Protein Residue Accessibility Predictor or MPRAP. In contrast to earlier methods MPRAP was not optimized to predict the accessibility in a particular region, but predicts the accessibility both inside and outside the membrane. This was simply done by using the complete TM-proteins in the training of the predictor. During the development of MPRAP different combinations of sequence derived input parameters were evaluated, see Table 2.

**Table 2:** Input parameters

Parameters	Specificity	Sensitivity	Accuracy	MCC
*Single inputs*				

AA	0.57	0.89	0.59	0.19
R4S	0.70	0.71	0.68	0.37
Zpred	0.60	0.70	0.60	0.19
Zcoord	0.56	0.75	0.56	0.12
*PSI*_*scr*_	0.72	0.70	0.69	0.39

*Combined inputs*				

R4S + Zpred	0.70	0.77	0.70	0.40
AA + Zpred	0.63	0.70	0.62	0.24
AA + R4S	0.70	0.78	0.71	0.41
AA + R4S + Zpred	0.71	0.77	0.71	0.43
*PSI*_*scr *_+ Zpred	0.72	0.70	0.70	0.40
*PSI*_*scr *_+ R4S	0.73	0.75	0.72	0.43
*PSI*_*scr *_+ R4S + Zcoord	0.73	0.73	0.71	0.43
*PSI*_*scr *_+ R4S + Zpred	0.74	0.74	0.73	0.44

*Different kernels*				

*PSI*_*scr *_+ R4S + Zpred (Linear)	0.72	0.74	0.71	0.41
*PSI*_*scr *_+ R4S + Zpred (Polynomial)	0.73	0.74	0.72	0.44
*PSI*_*scr *_+ R4S + Zpred (Radial basis)	0.74	0.74	0.73	0.44

*Different probe radii*				

*PSI*_*scr *_+ R4S + Zpred (1.4 Å)	0.74	0.74	0.73	0.44
*PSI*_*scr *_+ R4S + Zpred (2.0 Å)	0.78	0.82	0.74	0.45
*PSI*_*scr *_+ R4S + Zpred (2.0 Å/1.4 Å)	0.76	0.78	0.74	0.46

Different combinations of input parameters used to train a Support Vector Machine to predict surface accessibility in a two state alphabet. For each predictor the specificity, sensitivity, accuracy and the Matthew Correlation Coefficient for predicting buried residues in a binary alphabet is reported. The first five lines contain the prediction results using a single type of information, where AA is amino acid encoded using sparse encoding, R4S is the substitution rate calculated from rate4site scores, Zpred is predicted distance from membrane center, Zcoord is the real (not predicted) distance from the membrane center and *PSI*_*scr *_is PSIBLAST-PSSM. The next group of predictors was obtained using combinations of these inputs. The next two lines contain the results for two predictors using the optimal combination of inputs but other kernels than the radial-basis kernel. The last line is the performance of the final version of MPRAP, i.e. the one trained to predict absolute accessibility.

As shown above, there are two major factors that differentiate between exposed and buried residues in a membrane protein, substitution rate (conservation) and amino acid preferences. Exposed residues are evolving faster than buried residues both inside and outside the membrane, see Figure 2. The preference for certain amino acids to be buried or exposed is, however, dependent on the location relative to the membrane; Polar residues are more likely to be exposed outside the membrane than within the membrane, see Figure 1. Therefore, an optimal predictor needs to be able to determine the location of a residue in relationship to the membrane. This can be done either by explicitly providing this information as an input to the predictor or by assuming that the predictor indirectly will learn this.

During the optimization of MPRAP a two state binary classification of exposed and buried residues was used. During the testing 5-fold cross validation was used. The performance of the SVM was optimized by a systematic search of model parameters and the best performance was used. First, it was found that a window size of 9 seemed to be optimal and was therefore used for all different inputs. Several alternative methods to identify membrane and non-membrane residues, including topology predictions by OCTOPUS [21], were tested and the best performance was obtained using predicted distance from membrane center by Zpred [20]. It was also found that a slightly higher performance was obtained using a radial basis kernel than alternative kernels, see Table 2.

After these initial optimizations four different inputs to the SVM were examined, amino acid information (AA), predicted distance to the membrane center (Zpred), substitution rate (R4S) and PSSM information (*PSI*_*scr*_), see Table 2. All these inputs contain some information that is useful for predicting accessibility by themselves. However, the useful information in AA and Zpred is much lower (MCC = 0.19 and 0.19) than for R4S and *PSI*_*scr *_(MCC = 0.37 and 0.39). The good prediction performance of R4S is due to the strong correlation between accessibility and substitution rates, see Figure 2B. Since the PSSMs in similarity to R4S also contain conservation information this is most likely also the reason why input consisting of only *PSI*_*scr *_perform so well. However, accessibility is also dependent on the topology and the polarity of the residue [22]. Consequently, amino acid information (AA) in combination with R4S increased the prediction accuracy to MCC = 0.41, while using the PSSMs provides another slight increase to MCC = 0.43. The inclusion of a predicted distance from membrane center by Zpred [20] increased the performance only marginally. Interestingly, using the Z-coordinates from the structures provide slightly lower prediction accuracies than using the predicted Z-coordinates. This might very well be due to the observations that the most hydrophobic region not always correspond to the central membrane regions in the structures of membrane proteins [23].

### Performance of MPRAP

Finally the optimal input parameters found during the optimization were used to develop a predictor for real accessibility. The mean absolute error (18.4 Å) and Pearson correlation coefficient (0.58) of this predictor was found to be better than the other real value predictors, see Table 3. These values are comparable to the performance of predictors for soluble proteins [19,24].

**Table 3:** Performance of predictors using absolute numbers

Parameters	Cc	MCC	MAE
MPRAP	0.58	0.45	18.4
SABLE	0.40	0.29	21.9
*ASAP*_*mem*_	0.18	0.12	24.3
*ASAP*_*extmem*_	0.52	0.40	19.8
*ASAP*_*glob*_	0.41	0.32	21.6

Performance of the final version of MPRAP and other predictors that predict relative surface area.

Table 1 shows the performance of the MPRAP predictions after a transformation to a binary alphabet. The accuracy in the non-membrane region and membrane is comparable, see Figure 3 and Table 1, while the performance in the water-lipid interface region is slightly worse, perhaps due to greater structural variability in this region [25]. Anyhow, MPRAP outperforms all other predictors in the membrane core and in the water-lipid interface region, Table 1. In the non-membrane region MPRAP is outperformed by ACCPRO [12]. Further, MPRAP outperforms the combined predictor in all but the non-membrane region. Finally, we investigated the performance of three predictors on a dataset of water-soluble proteins, Table 1. As expected, ACCPRO and SABLE [19], methods optimized for such proteins performed well (MCC= 0.63 and 0.56 respectively). However, MPRAP performs on parity with SABLE (MCC = 0.55).

The reason for the improved prediction in the membrane region is probably mainly due to that MPRAP is trained on a larger dataset than earlier methods. Increasing the dataset size from 40 to 80 proteins increased the performance of the predictions from MCC = 0.36 to MCC = 0.45. This also suggests that one reason why ACCPRO outperforms MPRAP in the non-membrane regions might be because it was trained on a considerably larger dataset. However, including a larger set of soluble proteins into the training set of MPRAP did not improve the performance significantly (data not shown).

In order to investigate the predictive performance on different proteins in the dataset the proteins were divided into subgroups by the number of transmembrane regions, fraction of TM-residues and their multimeric state. In all these subgroups the performance was similar (MCC = 0.42-0.46). Thus, no particular type of membrane proteins was identified where MPRAP performed significantly better or worse for.

Above, a van der Waals radii of 1.4Å, mimicking the size of a water molecule, was used to calculate the accessibility of membrane proteins. However, within the membrane, a more realistic choice for calculating the accessibility might be to use a larger probe (2.0 Å) to mimic a *CH*_2 _group. Therefore, at the end three different versions of MPRAP were developed, using probes of 1.4Å, 2.0Å or a combination of these. They all perform similar, see Table 3, and the probe size of 1.4Å is set as default.

The most important improvement over earlier predictors is that MPRAP is the first accessibility predictor that shows an acceptable prediction quality in all regions of membrane proteins. This is obtained without any pre-processing. We believe this is predominantly the result of careful selection of an appropriate training set consisting of entire membrane proteins.

### MPRAP can identify some erroneous protein structures

One application of accessibility predictors is model quality assessment of a protein model. We may assume that a correct model should have higher agreement with predicted accessibility than an incorrect model. Recently, three structures of MsbA and two of EmrE have been retracted from PDB [26]. It has been shown that comparison between the predicted versus the "true" distances from the membrane center (Z-coordinates) can be used to indicate that some of these models are problematic [27]. Here, we wanted to examine if MPRAP can identify the problems with these models. All five structures were found to have low (almost random) agreement between observed and predicted accessibility, Table 4. In contrast the new, presumably correct, structures of MsbA were found to have a higher agreement (MCC > 0.6). This indicates that MPRAP might be useful for model quality assessment, at least in some cases.

**Table 4:** Assessing the quality of protein structures

PDB	Protein	Resolution	Accuracy	MCC
1s7bB	EmrE	3.8	0.51	-0.09
2f2mA	EmrE	3.7	0.48	-0.19
1jsqA	MsbA	4.5	0.55	-0.06
1pf4A	MsbA	3.8	0.55	0.10
1z2rA	MsbA	4.2	0.63	0.26

1l7vA	MsbA	3.2	0.81	0.64
2hydA	MsbA	3.0	0.85	0.69

Agreement between predicted and structurally derived accessibility on six PDB-structures. The four structures at the top are published structures that have been removed from the database due to discovered anomalies. The two structure at the bottom are recent structures of proteins from the same protein families that are present in PDB.

### Some interaction surfaces can be identified

Many transmembrane proteins consist of several peptide chains positioned in a complex. During the calculation of accessibilities, all chains in a complex were included, i.e. if a residue is located in the interface between two peptide chains it was classified as buried. However, in some cases the biological unit of the full complex is not completely known and therefore it might be of interest to identify interface areas. Here, we define interface residues as residues that are buried (<25% accessible surface area) in the full complex, but exposed in the single protein chain. Among the interface residues 50% are predicted as exposed and 50% as buried, Table 5, clearly distinguishing this group of residues from those exposed in the complex or buried in the single chain.

**Table 5:** Identification of interface residues

	***B***_***MPRAP***_	***E***_***MPRAP***_
Buried	74%	26%
Interface	51%	49%
Exposed	21%	79%

Fraction of residues predicted by MPRAP to be buried (<25% accessibility), *B*_*MPRAP*_, or exposed, *E*_*MPRAP*_, among the buried, interface and exposed residues.

**Figure 4:**
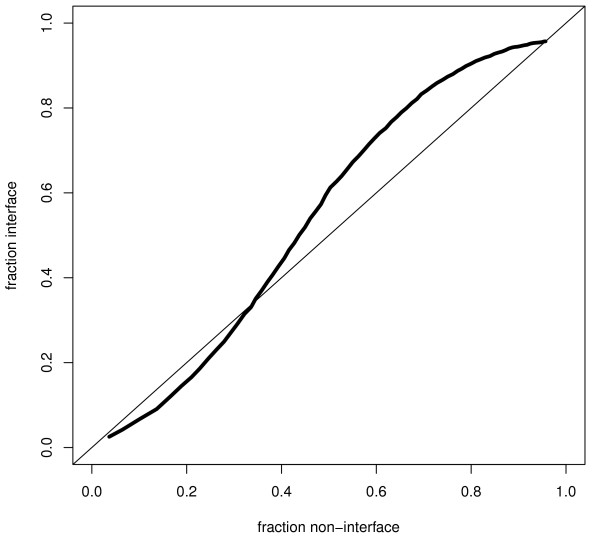
**Identification of interface residues**. Identification of interface residues among residues exposed in a single protein chain. For all these residues MPRAP was used to predict its accessibility. At a given MPRAP cutoff the fraction of all interface residues predicted to have accessibility less than the cutoff is plotted against the fraction non-interface residues above this cutoff.

This indicates that MPRAP could be used to suggest possible interaction sites in membrane protein structures where some interaction partners are missing. If MPRAP predicts that some exposed residues should be buried this might indicate that they are involved in an interaction with another protein chain. In line with this idea MPRAP was applied to all exposed residues in the single protein chains. Then it was assumed that residues predicted to be buried belonged to an interface area, see Figure 4. It was found that among the sites predicted to have less than 30% accessibility 80% were true interface residues.

## Conclusions

One prominent feature of membrane proteins is that their surfaces face three distinct environments; a hydrophobic lipid environment inside the membrane, an interface environment and a hydrophilic water environment outside the membrane. Here, we have analyzed the properties of exposed and buried sites in a set of membrane proteins of known structures. As expected, we found that exposed sites are different inside and outside the membrane. In contrast, residues at buried sites are more similar but also on average more hydrophobic inside the membrane than outside. Further, in all regions exposed residues are less conserved than buried residues.

The problem of predicting accessibility of individual residues is a well-studied problem for non-membrane proteins but less so for membrane proteins. We found that all state-of-the-art predictors for surface area are optimized for one of the environments and therefore perform poorly in the non-optimized environments. To circumvent this problem we included complete membrane proteins in the training set and developed a new predictor, the Membrane Protein Surface Accessibility Predictor (MPRAP). The new predictor performs well both inside and outside the membrane. Further, MPRAP is better than the combination of two specialized predictors. This shows that MPRAP is capable of recognizing the fact that there are different preferences for exposed sites within and outside the membrane, i.e. it can adjust the predictions depending on the relative localization to the membrane. One reason why this is possible is the strong correlation between exposure and conservation.

## Methods

The creation of the dataset started from 136 *α*-helical TM protein structures containing 601 polypeptide chains with TM segments from OPM [17] in April 2008. Poly-alanine chains, theoretical models and obsolete entries (as defined by PDB) were excluded. In addition, fragments (1D6G, 1ORS, 2AHY, 1R3J, 1S5H), very low-resolution structures (>3.9) A, 1IFK, 2BG9) and structures with secondary structure or membrane boundary problems (2QFI, 1ORQ, 2A01, 1YEW) were removed.

Uniprot sequences corresponding to the remaining sequences were used to search for homologs by running three rounds of PSI-BLAST [28] using a conservative E-value cutoff of 10^-5 ^against uniref90 [29] from November 2007. Chains with less than four identified homologs, mostly of short transmembrane proteins were removed in a second filtering step, to enable the use of substitution rate information. The structure of highest resolution from each OPM family was chosen as a representative. Blastclust [28] was used to further reduce the number of chains. Default parameters were used with a sequence identity cutoff set to 20%, no E-value cutoff and using a default length cutoff of 0.9. The obtained sequences were also checked afterwards by running blast for each protein on the new dataset and no pairs showed an identity over 20% independent of length. Further, all chains from the same OPM superfamily were put in the same cross-validation group during the SVM training (see below). The final selection of protein chains and their grouping into the five cross validation groups are provided as supplementary data [Additional file 1].

### General analysis

As in our previous studies [11,25,30], all proteins were oriented so that the predicted membrane center was located at the X-Y plane, thus the proteins could easily be studied as a function of the Z-coordinate. Here, the OPM method was used to find orientation [17]. The Z-coordinates were used to classify all residues into three main groups: non-membrane (Z > 22Å), lipid-water interface (10 Å < Z < 22Å) and membrane core (Z < 10Å). In addition, membrane boundaries as defined by OPM were used. The classification into different groups was just used for evaluation purpose and not as input for predictions. The final dataset contained 21,624 residues, with 5,565 in the core, 7,114 in the lipid-water interface and 8,945 in the non-membrane region. The residues were grouped after physico-chemical similarity, using the biological hydrophobicity scale [31] into hydrophobic [A, F, I, L, M, V], weakly polar [G, Y, W, C, S, T] and strongly polar [D, E, K, R, H, N, P, Q]. In total 9,931 hydrophobic, 6,258 weakly polar and 5,435 strongly polar residues were found.

The amino acid substitution rates were estimated as described in [11], by using the PSI-BLAST derived multiple sequence alignments (mentioned above) as input to rate4site [32]. The residue values from rate4site are normalized for each protein individually, by subtracting the average substitution score and dividing by the standard deviation.

Surface accessibility was calculated by Naccess 2.1.1 [33] using probe sizes of 1.4 and 2.0 Å. The complete protein-structure (including prosthetic groups and other polypeptide chains) was used, but lipids and water were removed. The relative surface area (RSA) was obtained directly from Naccess, where the accessible surface area of a residue is normalized by an extended A-X-A tri-peptide conformation. All accessibility values were also converted into a binary state alphabet, with residues less than a certain cutoff as buried and all others as exposed. The cutoffs were optimized to give highest MCC during the evaluation independently for each method. This resulted in an approximately equal frequency for the two states. In addition a pre-compiled subset of 1,607 sequences from the entire PDB with maximum resolution, R-factor and mutual sequence identity of 1.6 A, 0.25 and 20%, respectively, was downloaded in January 2010 from the PISCES database server [34]. Chains predicted to have at least one transmembrane region were removed. The accessibility of the remaining and assumed water-soluble proteins was calculated in the same way as the membrane dataset.

### Development of MPRAP

The ability to predict the relative solvent accessibility was investigated using support vector machines implemented in the svmlight package [35]. All experiments were performed using a 5-fold cross-validated training of support vector machines, with approximately equally many proteins in each subgroup. For each set of input variables three different kernels (linear, polynomial and radial basis) were tested. For all kernels a grid search was used to find the optimal parameters. For all kernels the trade-off between training error and margin (the -C parameter in svmlight) was varied between 0.25 and 50 in steps of 0.25. For the polynomial kernel three exponents were used, 2, 3, and 4, and for the radial basis function values of the parameter were tested between 0.0005 to 0.05. In most cases the radial basis kernel was found to be optimal.

A number of sequence-derived parameters were tested as inputs to the SVM, including amino acid frequencies from PSI-BLAST, substitution rates, and predicted distance from membrane center, see Table 2. Different sizes of a symmetric window were investigated and a window size of 9 was found to be optimal. It was also found that including the PSI-BLAST PSSM values directly was superior to amino acid frequencies or normalized PSSM values.

A number of ways of distinguishing TM and non-TM residues, including topology predictions, were tested. The best performance was obtained using predicted distance from membrane center by Zpred. In an attempt to increase the performance in the lipid-water interface region, residues were classified based on the predicted distance from membrane center into two (non-membrane, membrane) or three (non-membrane, lipid-water interface and deep membrane core) different groups. Thereafter, SVMs were trained separately for each group. However, this did not result in any improved performance (not shown). Another attempt to increase the performance in the non-membrane region was to add a varying number of water-soluble proteins to the training set. However, no attempts in this direction improved the performance significantly and therefore no soluble proteins were included in the training of MPRAP. Two different probe radii were used for accessibility calculations (see above). A probe radius of 1.4 Å might be ideal for water-soluble region (to mimic water) and a probe radius around 2 Å might be better for membrane region (to mimic a CH2-group). The performance of MPRAP trained for 1.4 Å, 2.0 Å, or a combination of 1.4 (outside the membrane) and 2.0 (inside the membrane) resulted in very similar performance.

For evaluation purposes the real value predictions were transformed to a binary classification in a similar way as the training values. Values below a cutoff were in this step classified as buried and others as exposed. This procedure resulted in an approximately equal amount of buried and exposed residues. For evaluation purposes buried residues that were predicted to be buried were assigned as true positive (TP), buried residues predicted to be exposed as false negatives (FN), exposed residues predicted to be exposed as true negatives (TN) and exposed residues predicted to be buried as false positives (FP). From these numbers the following measures were calculated:

• 

• 

• 

• 

Here, MCC is the Matthems Correlation Coefficient [36]. Additionally, for real value predictions mean absolute error (MAE) and Pearson correlation coefficients (Cc) were calculated.

### Benchmarking surface area predictors

Zpred, TMX and the ASAP-predictors were run directly from the web-servers, while ACCPRO and SABLE were run locally. The predictors have used slightly different probe sizes, different programs and methods for calculation of accessibility. Therefore, the exact definition of the predicted feature differs. Both ACCPRO and TMX predict accessibility in a binary state alphabet at approximately equal frequency, while MPRAP, SABLE, *ASAP_glob _*and *ASAP_mem _*predict real accessibility values. Therefore, to make as fair comparison as possible the real values were transformed to binary states. The cutoffs used for transformation were optimized separately for best performance, mostly resulting in approximately equal frequency of buried and exposed states. The cutoffs were set to 20% for ASAP, 15% for SABLE and 25% for MPRAP. Thereafter, the binary states derived from the predictors were compared to the binary states derived from the known NACCESS values (see above). For the combination of TMX and ACCPRO, Zpred was used to decide which predictor to trust for each residue. If a residue were predicted to be closer than a certain cutoff from the membrane center TMX was trusted, and if not ACCPRO was used. The cutoff was optimized to be 12.5 Å.

### Assessing quality of protein structures

Agreement between predicted and structurally derived accessibility was tested on six PDB-structures downloaded from PDB: 1S7B, 2F2M, 1JSQ, 1PF4, 1Z2R, 1L7V and 2HYD. Four of the first five were marked as obsolete in PDB. The accessibility values were calculated for the full complexes using the same procedure as in the training dataset for MPRAP (see above). The values that were considered for evaluation are from the A subunit of the full (homo oligomeric) complex. The cross-validation group containing 2HYD where left out in the training set when assessing the quality of 1JSQ, 1PF4, 1Z2R, 1L7V and 2HYD.

### Sites at polypeptide chain interfaces

Residues at interaction surfaces, *I*_*NACCESS*_, were identified as all residues that have higher accessibility than 25% in the single chain structure and lower than 25% in the full complex.

A residue was predicted to be in an interface if it is exposed in the single chain and MPRAP predicts it to have lower accessibility than a certain cutoff. The fraction of interface residues detected among all sites that are exposed in the single chain structure were evaluated for all sites that were exposed in the single chain.

### Data analysis and visualization

The molecular illustrations were created with PyMol (DeLano, W.L. The PyMOL Molecular Graphics System (2002) DeLano Scientific, Palo Alto, CA, USA). The remaining figures were generated in R [37].

## Authors' contributions

All authors conceived the project and design. KI and SC prepared the data and training of the predictor. KI and AE analyzed the results and wrote the paper. All authors read and approved the document.

## Supplementary Material

Additional file 1 **Dataset**. The dataset used for training of MPRAP. The first column in each line contains the name pdb code of a protein followed by the chain identifier in the fifth column. The second column describes in which cross-validation group (A-E) each chain belongs. Click here for file
